# Probabilistic Perception, Empathy, and Dynamic Homeostasis: Insights in Autism Spectrum Disorders and Conduct Disorders

**DOI:** 10.3389/fpubh.2014.00004

**Published:** 2014-01-27

**Authors:** Jean Marc Guilé

**Affiliations:** ^1^Groupe de Recherches sur l’Analyse Multimodale de la Fonction Cérébrale, INSERM 1105, Université Picardie Jules Verne, Amiens, France

**Keywords:** empathy, homeostasis, infant, autism spectrum disorders, conduct disorder

## Abstract

Homeostasis is not a permanent and stable state but instead results from conflicting forces. Therefore, infants have to engage in dynamic exchanges with their environment, in biological, cognitive, and affective domains. Empathy is an adaptive response to these environmental challenges, which contributes to reaching proper dynamic homeostasis and development. Empathy relies on implicit interactive processes, namely probabilistic perception and synchrony, which will be reviewed in the article. If typically-developed neonates are fully equipped to automatically and synchronously interact with their human environment, conduct disorders (CD) and autism spectrum disorders (ASD) present with impairments in empathetic communication, e.g., emotional arousal and facial emotion processing. In addition sensorimotor resonance is lacking in ASD, and emotional concern and semantic empathy are impaired in CD with Callous-Unemotional traits.

Autism spectrum disorders (ASD) and conduct disorders (CD) are both of public health concern. Prevalence for ASD reaches 0.7% in general population (1), while CD bears high social burden, especially CD with high callous-unemotional traits (CD-HCU), with elevated rate of violent crime. Both disorders are associated with social maladjustment and impaired communication and empathy. Within an evolutionary perspective, empathy is an adaptive response to the continuously challenging social environment and an efficient means to reach homeostasis. With respect to key concepts such as homeostasis, perception, and synchrony, the present article will review the development of empathy in typically-developed (TD) youth before addressing empathy impairments pertaining to ASD and CD-HCU.

## Dynamic Homeostasis and Empathy

Homeostasis, which is marked by extreme instability according to Cannon (2), cannot be obtained without exchanges with the environment. Disequilibrium is a basic characteristic of human beings. Humans cannot survive on their own and must engage in dynamic exchanges with their physical and human environment. Revisiting Cannon’s definition of homeostasis, Arminjon et al. (3) appropriately stressed that homeostasis is not referring to a permanent and stable state but instead results from the interaction of conflicting forces. The newborn’s innate propensity to communicate with other humans allows for searching the biological, cognitive, and affective inputs required for his/her development. Empathy encompasses the cognitive mechanisms, which provide security and cohesiveness among these conflicting interactions. Such mechanisms have been traced back in primates and other mammals (4, 5). From an evolutionary standpoint, empathy is an adaptive mechanism to help secure individuals’ development and survival.

Empathy refers to the intuitive access to others’ subjective experience (6). It denotes the individual’s capacity to understand others’ intents and experience their feelings. Empathy is currently conceived as a cognitive capacity, which supports individuals’ ability to behave with respect to socially relevant information. Empathy is not a unitary function and encompasses three processes, which successively appear as the child matures: procedural/implicit, semantic, and biographical empathy. Procedural empathy refers to the innate and non-conscious capacity to resonate with others’ emotional states. Procedural empathy itself encompasses three components seen in neonates and primates: sensorimotor resonance, also called motor contagion, emotional arousal/contagion, and empathetic concern. Sensorimotor resonance supports behavioral (7) and neural (8) mirror activations. Emotional arousal is the emotional contagion resulting in similar emotion being aroused in the observer as a response to the expressed emotion of another (8). Empathetic concern is an “other-oriented emotional response congruent with the perceived welfare of someone in need” (8). It results from the attachment system, which develops between the infant and his/her primary caregivers (9). Semantic empathy parallels language development and expresses connection between words, meaning, and emotion. All these processes take place before the installation of theory of mind when the child turns four. Biographical empathy emerges later in life and corresponds to the interweaving of personal experience with feelings and words, together with a capacity to bridge with others’ experiences.

Over the past decade, electrophysiological (ERP) and neuroimaging studies, especially on others’ pain perception, have shed light on the neural underpinnings of empathy. Because emotions are expressed through facial mimicry, the neural circuitry of empathy lies at the crossroads of networks involved in attention, emotional arousal, face perception, intent recognition and self-awareness (agency). Pain ERP studies have elicited a very early response occurring at 60 ms after exposure to a painful visual stimulus (10, 11). High-density ERP with brain source analysis techniques identified the signal source in the right temporo-parietal junction (TPJ), a cortical area bridging visual and attentional cortical pathways with amygdala and prefrontal networks. Such activation discriminated between intentional and accidental harmful behaviors. Previous neuroimaging studies conducted by the same group have identified TPJ as a pivotal network in discriminating self and other actions (agency) (8). Later potentials have been identified around 120 ms after stimulus onset over the amygdala or the anterior cingulum depending on the study. This 120 ms negativity is usually viewed as arousal response combining attentional and emotional reactiveness. Of note, negative potentials occurring around 170 ms after stimulus onset are specifically associated with face processing (12). Later activations denote prefrontal recruitments and contributions from top-down regulation mechanisms. In sum, ERP studies point to the earliness of cognitive responses triggered by the intent and emotional value associated with the stimulus, even before its shape and identity are fully disclosed.

Neuroimaging studies on empathy for pain provide results complementary to ERP data. FMRI studies have consistently stressed the association between empathy and activation of regions such as the anterior insula, anterior cingulate, and amygdala. On the contrary, the involvement of sensorimotor areas is still unclear. Studies diverge on whether pain direct and vicarious experiences recruit the same brain sensorimotor and limbic networks. Observing pain in someone else is associated with partial activation of the pain matrix recruited when experiencing oneself pain, namely anterior insula/fronto-insular cortex and the anterior cingulate cortex (13). Activation of the sensorimotor and somatosensory cortices seemed to be restricted to direct painful experiences. Recent studies, however, have shown the activation of sensory cortices (14) and motor cortices (15) in perception of other’s pain. These findings lent support to the existence of a separate although empathy-related network responsible for sensorimotor contagion. In sum, apart from subcortical structures as amygdala, empathy networks encompass prefrontal and anterior cingulate cortices as well as interconnected areas like insula and TPJ. These cortical networks indirectly receive information from visceral sensory systems about internal homeostasis, which impacts their activation (16).

## Probabilistic Perception and Parent–Infant Synchrony

Infant’s early empathy behaviors rely on non-conscious and innate procedural/implicit cognitive processes, which themselves depend on the perception of auditory and visual social cues. Such cues attest to the presence of care givers in the infant’s environment. Although sensory inputs are basically continuous and ambiguous, infants as young as 8-month-old have been shown to be capable of word discrimination. This capability seems to result from statistical learning processes (17). Infants can detect within the continuous voice flow statistical patterns (e.g., pairwise association between letters and syllables) that serve as a cue to word boundaries (18, 19). Each sensory input gives rise not only to one interpretation but also to a large range of inferences regarding the true state of the perceived environment (20). This probabilistic perception allows for building up an internal model of rhythms, objects, and people, which are the source of sensory inputs. This internal model is subsequently modified according to subsequent sensory inputs.

In naturalistic settings, subsequent inputs are provided through interactions between parents and infant. Parents’ vocalizations, called infant-directed-speech (IDS) or motherese (21), trigger the infant’s vocalizations, which in turn lead to parental responses. As observed in family home videos, parents and infant interactions gradually evolve toward a synchronous pattern of mutual attention, speech, and gestures without any conscious intent from the participants (22). Following Delaherche et al. (23), synchrony could be viewed as the dynamic and reciprocal adjustment to the temporal structure of interactive behaviors and emotions between communication partners, e.g., mother and infant smiling at each other and babbling together during feeding time. Synchrony, which constitutes the core phenomenon of empathy, is probably the main factor sustaining probabilistic perception and learning. Infants identify probabilistic patterns within verbal interactions with their parents. How such patterns of co-occurring vocal sounds get selected and merged with the infant’s internal model of the perceived environment? One could stress the frequency of these co-occurrences. Another explanation would be that the parental synchronous response operates as a validation of the infant’s probabilistic perception mechanisms. Synchrony induces a selection within the perception inferences aroused by sensory inputs. Consequently, it enhances the formation of shared representations of the perceived verbal environment between infant and parents. It could be one of the mechanisms by which social interaction affects implicit learning (24). According to this hypothesis, synchrony would foster language acquisition in non-verbal infants.

Synchrony not only implies cognitive but also bodily processes. Damasio (16) emphasized the involvement of brain–body pathways, including autonomous nervous system, which shape composite and dynamic maps of the body’s state from moment to moment. Biological expressions of synchrony have been consistently demonstrated through vagal tone modulation and oxytocin secretion during mother–infant interactions (25, 26).

In keeping with the framework of dynamic homeostasis, probabilistic perception theory gives to perception, a definite characteristic of incompleteness and disequilibrium. Such mechanisms require, in turn, appropriate response from the environment to achieve adequate perception. Homeostasis is achieved through synchronous non-conscious exchanges between parents and infant. Synchrony is the common basic mechanism, which underlies mirror mechanisms observed at neural and cognitive levels in neuroimaging and ERP studies on empathy.

## Empathy and Dynamic Homeostasis in Typically Developed Infants

Decety and Svetlova (8) put together ontegenetic and phylogenetic perspectives to set up an evolutionary model of the development of empathy. Several adaptations of cognitive processes, both procedural/implicit and explicit, have been added to the social brain across evolution. Rather than ended up with a well coordinated structure of empathy mechanisms, it seemed that evolution led to a “patchwork of additions” encompassing various processes, each with a distinct evolutionary history and neural networking, and a parallel although interdependent functioning. Among the procedural mechanisms, self-awareness (agency), which allows for discriminating between self-generated actions and others’ behaviors, seems to be human-specific. Other human-specific mechanisms, like explicit processes, which prevent emotional upsets and regulate emotional concern capacities, operate gradually over the child development according to caregivers inputs. Such a complex and interactive construct allows for a wide range of flexibility and adaptive responses to environmental challenges.

Synchrony, which plays a pivotal role in developing empathy, stems from early interactions *in utero* and all across infant development since birth. It affects the infant’s first perceptions whatever the different sensory modalities and triggers unimodal and crossmodal parental responses, although studies report mainly on auditory and visual social stimuli. Premature babies present syllabic discrimination capacities and voice recognition by the 28th week of gestation (27) and the fetuses show indication of maternal voice recognition in by the 34th week of gestation (28). A pattern of synchronous parent–infant vocalizations has been observed in family home videos with TD infants showing the impact of synchrony on infant language development (21). During second semester, the parents’ motherese (vs. other speech) is significantly followed by more infant’s vocalizations. In a recent neuroimaging study, synchrony was associated with mother’s brain activations in the dorsal anterior cingulate (dACC), cuneus, fusiform, supplementary motor and TPJ cortices, dACC being the most sensitive area to the mother’s synchronous response to her infant (29).

With regard to visual processing, first-trimester-old neonates demonstrate preference for the upper part of human faces and gaze-following capacities. By 6-months of age, infants allocate increased attention to angry faces with direct eye gaze as well as to fearful faces looking toward objects and objects that are gaze-cued by a fearful face (30, 31). Behavioral sensorimotor resonance, the capacity to imitate complex specific facial mimicry, has been shown in newborns as early as a few days-old infants (7). This automatic mimicry disappears by 3-months of age as these implicit mechanisms are replaced by more adaptive responses proper to the particular affective context of interactions between the infant and his/her caregivers. Twelve-month-old infants tend to read other humans’ intentions beyond the observed behavior and attribute discriminate goals to others (32, 33). These results, pointing to early forms of self-awareness, are consistent with experiments demonstrating the infant’s early discriminate reactivity to his/her own recorded cry in comparison to the cry of another baby (34). More elaborated and explicit self-awareness/agency processes later develop in the second year of life alongside perspective-taking increasing capabilities (35) and spatial abilities (36).

Taken together, it seems that neonates are cognitively equipped to detect environmental threats and others’ intents. On the opposite, their capability to respond adaptively to such inputs depends on the context, especially the ability of their human environment to decode in return their emotion and intent to perform actions. As a consequence, automatically elicited implicit detection and response processes tend to vanish over the course of infant development to be replaced by contextually developed more adaptive mechanisms with regard to the specific context of the infant’s relationships with his/her caregivers. At birth perceptual and neural systems are wired to be sensitive to social information, and with age prefrontal and limbic networks get more matured and connected, allowing for more contextually and experience-driven interactions (8). These developmental processes allow for achieving proper dynamic homeostasis.

## Empathy and Dynamic Homeostasis in Autism Spectrum Disorders

**Figure 1:**
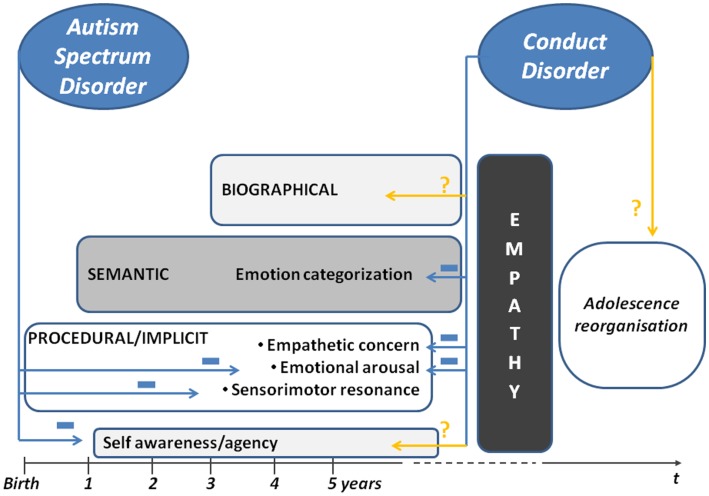
**Development of empathy in early childhood**.

Autism spectrum disorders are associated with major impairments in social communication and adaptation, as well as self-awareness/agency immaturity (37). Disturbances and alternative cognitive processes affecting perception and empathy, especially procedural systems, have been identified over the past decades. These cognitive characteristics put the ASD child’s homeostasis achievements at stake (Figure 1). The child often asks for repetitive activities and interactions while relying mainly on personal inputs, rather than bridging with others, to maintain homeostasis.

In keeping with seminal studies of Saffran et al. (18), research has been conducted to explore probabilistic perceptual mechanisms used in ASD (38). Individuals with ASD are less susceptible of perceptual illusions and less influenced by acquired expertise on perceived objects. Since perception in ASD is less constraint by previously memorized organization of sensory inputs, it might slow down new acquisition and adaptation in ordinary social context while providing more perceptual accuracy in unexpected situations.

Spontaneous mimicry, observed in TD infants, is lacking in ASD (39) whereas voluntary mimicry is often preserved (40). Sensorimotor resonance, as measured by spectral EEG (41) and ERP (15, 42) is impaired in ASD. In a study on self and other pain perception, in contrast to TD participants, adults with Asperger Disorder did not show any modification of motor-evoked potentials corresponding to the muscle vicariously affected by pain (15). Since mimicry and facial emotion discrimination implied recruiting somatosensory-related cortices (42), impairment in facial emotion detection observed in ASD could be related to a lack of activation of sensorimotor cortical networks.

Behavioral as well as neuroimaging studies on fearful facial emotion have elicited an abnormal pattern of emotional arousal. Individuals with ASD, adults (43), and children (35), gazed more often away from than toward the eyes in comparison with a TD group. ASD adults showed increased amygdala activity when the eyes included in the fearful facial stimulus appeared at the initial location of the fixation point, compared with initial fixation at the mouth and compared with the TD individuals (43). In the experiment, increased initial amygdala activation was associated with subsequent gazing off the fearful stimulus. This reaction pattern differs from TD infants whose attention is attracted by fearful faces.

At first glance, semantic empathy seemed to be less impacted in ASD, since behavioral studies using facial emotion categorization tasks did not yield significant differences between ASD and TD groups, even when using multimodal sensory inputs (44). However, significant differences were retrieved when the experiments imply a sequential presentation of the stimuli, thus involving a working memory component (45). This result is consistent with observations from Pellicano and Burr (38) on visual expertise. In addition, neuroimaging studies stressed the alternative recruiting strategies observed in ASD adults and children. In comparison to TD participants, ASD children showed less activation in fusiform gyrus (46), an area which would undergo cortical thinning later in child development (47). It suggests that ASD children use more configurational and less emotion-driven information than TD while processing facial emotion (46).

A wide area is offered to further researches, including the exploration of synchronous social processing between ASD children and their caregivers in order to assess the impact of synchrony on social learning. Analyses of naturalistic home videos with infants, who later developed ASD already showed an increase in parental stimulation and IDS during the second semester (48). Clinical observations tend to support that attachment processes, and consequently empathetic concern, the third component of procedural empathy, are relatively preserved despite some impairments in the perception of others’ needs. Therefore given procedural empathy impairments, homeostasis dynamics in family affected with ASD would be modified, offering the child with more adaptive parental homeostatic responses.

## Empathy and Dynamic Homeostasis in Conduct Disorders with High Callous-Unemotional Traits

Conduct disorders with high callous-unemotional traits (CD-HCU), also called psychopathy, is a CD subgroup with a restricted emotional expression, callous lack of regard for others, and severe violent behavior against people (49). Although the complete syndrome appears in childhood with later reorganization related to the evolution of peer-relationships in adolescence, precursors can be traced back to infancy [for a review see Ref. (50)]. A few studies, exploring empathy, have been conducted on pain detection and facial emotion processing.

Sensorimotor resonance, as measured by attenuation of the μ band (8–12 Hz in central region), appeared unaffected in both CD-HCU and CD-LCU male adolescents while observing pain in others (51). However, no frontal N120 increase was observed in CD-HCU, indicating attenuated emotional arousal compared to TD and CD-LCU controls. While processing pain, CD-HCU participants exhibited significantly less activation in the ventromedial prefrontal cortex and lateral orbitofrontal cortex, but greater activation in the insula, compared with CD-LCU (52). Low emotional arousal was equally observed in Adolescents with CD-HCU, while processing fearful faces compared to CD-LCU and TD controls (53). Clinical observations indicate that empathy concern, another procedural empathy process which is related to attachment, is impaired. Parental deficits in empathy, which were known to contribute to CD in their offspring (50, 54), might impede the development of a proper attachment system and consequently mediate the lack of emotional concern observed in CD-HCU children. Deficits in semantic empathy have been consistently found in CD, namely fear-recognition deficit and hostility bias in labeling facial emotion (55, 56) [for a review see Ref. (57)]. In addition, impairments in biographical empathy would be expected, given the association between early life adversities and child maltreatment, and CD (58).

In sum, several components of procedural empathy are affected in CD-HCU compared to CD-LCU and controls. Emotional arousal for pain and fear is decreased as well as empathetic concern. Semantic empathy is impaired with a propensity to dismiss fearful faces and label neutral faces as hostile (Figure 1).

## Implications for Treatment

Recent pilot studies on short-term remediation programs addressing specific ASD empathy impairments yielded promising results with measurable improvements in face processing skills of ASD children (59). Given the decrease in emotional arousal and semantic empathy for fear observed in psychopathy, computerized cognitive remediation has been proposed to CD-HCU youths, training them to focus on the eyes of fearful faces. Pilot studies showed that such cognitive remediation programs can ameliorate fear-recognition deficits (53, 56).

## Conclusion

If TD neonates are fully equipped to synchronously interact with their human environment, ASD and CD-HCU present with disturbances in emotional arousal and facial emotion processing. In addition sensorimotor resonance is lacking in ASD, and emotional concern and semantic empathy are impaired in CD-HCU. These impairments prevent ASD and CD-HCU to achieve proper homeostasis. Further research is needed for exploring probabilistic perception mechanisms in these clinical populations on the one hand and, on the other hand, investigating patterns of co-activation between mother and infant with neuroimaging and electrophysiology.

## Conflict of Interest Statement

The author declares that the research was conducted in the absence of any commercial or financial relationships that could be construed as a potential conflict of interest.
